# Relative Age Effect in 14- to 18-Year-Old Athletes and Their Initial Approach to This Effect—Has Anything Changed Over the Past 10 Years?

**DOI:** 10.3389/fspor.2021.622120

**Published:** 2021-03-23

**Authors:** Ronnie Lidor, Zohar Maayan, Michal Arnon

**Affiliations:** The Academic College at Wingate, Wingate Institute, Netanya, Israel

**Keywords:** birth date, individual sports, team sports, youth sport programs, sport policy

## Abstract

One of the environmental variables associated with early talent development and the achievement of a high level of proficiency in sport is the relative age effect (RAE). The purpose of our study was threefold: (a) to calculate the RAE in young Israeli athletes (ages 14–18 years); (b) to examine how the athletes perceived this effect, if the effect indeed exists; and (c) to compare the RAE findings of this study with those of two previous studies on elite male (Lidor et al., 2010) and female (Lidor et al., 2014) Israeli ballplayers. Participants in the current study were 1,397 athletes (390 females and 1,007 males) who competed in five individual (gymnastics, judo, swimming, tennis, and track and field) and five team (basketball, soccer, team handball, volleyball, and water polo) sports. Data on the RAE, as well as on a number of aspects associated with this effect as perceived by the athletes, were collected *via* two closed questions. Data analyses showed that the RAE was found to be significant among the male athletes in four sports—swimming, basketball, soccer, and team handball; those who were born early in the year had a higher representation in these sport programs. However, this effect was not found to be significant in the female athletes. Most of the female and male athletes did not think that their birth date influenced their athletic success. However, a large portion of those who were born in the first quarter of the year (Q1) and the second quarter of the year (Q2) among the male athletes felt that they exhibited stronger abilities in the sports program compared to their peers who were born in the third and fourth quarters of the year (Q3 and Q4, respectively). The data of the current study provide additional support for the use of an “open door” approach to accepting children to sport programs by policymakers and coaches in Israel.

## Introduction

The relative age effect (RAE)—one of the environmental variables associated with early talent development and with achieving a high level of proficiency—has been extensively studied in the domain of sport. For example, a recent edited book entitled *Relative Age Effects in Sport—International Perspectives* (Dixon et al., 2020) discusses various aspects related to the effect's contribution to attaining excellence in sport. The term RAE reflects the asymmetrical distribution of athletes based on their birth date relative to an arbitrary cutoff date. For example, athletes who are born soon after a determined cutoff date have a higher representation in elite sport leagues compared to athletes born later (see Barnsley et al., 1985; Côté et al., 2006; MacDonald and Baker, 2013; Hill and Sotiriadou, 2016). Presumably, this effect can be an influence on the recruitment of individuals in sports, as well as on how they develop their athletic abilities/skills.

The RAE implies that individuals who are relatively older than their peers in a given cohort/year, or that their birth date is closer to the cutoff date for their age group classification, are more likely to reach higher athletic achievements (see Thompson et al., 1991; Musch and Grondin, 2001; Côté et al., 2006; Wattie et al., 2008; Schorer et al., 2009; see also Cobley et al., 2009; see Baker et al., 2009; MacDonald and Baker, 2013; Smith et al., 2018, for extensive reviews on the RAE). For example, a number of studies on elite individual and team sports found that athletes who were born early in the competition year had a higher representation than those who were born late in that year (e.g., Baker and Logan, 2007; Schorer et al., 2009). In most studies, two general explanations for the RAE in elite athletes were proposed: (a) the older athletes are more experienced than younger athletes in various motor–physical abilities, such as balance, coordination, speed, and strength, and therefore their performance of these sport skills is enhanced, and (b) the older athletes in their cohort/year are more likely to be selected to better teams and therefore are provided with more advanced guidance and training compared with younger athletes (see, e.g., Cobley et al., 2009; MacDonald and Baker, 2013).

In order to explain conceptually the phenomenon of the RAE, researchers proposed a number of RAE theoretical frameworks. For example, Hancock et al. (2013) discussed three principal social agents that have the potential to influence RAE: parents, coaches, and athletes. In their model, one sociological/psychological theory was proposed for each social agent in order “to explicate the genesis, perpetuation, and amplification of RAEs in sport” (Hancock et al., 2013, p. 631). The three discussed theories were the Matthew effect (social agent: parents), the Pygmalion effect (social agent: coaches), and the Galatea effect (social agent: athletes). The relationships between the three social agents will determine the magnitude of the existence/nonexistence of the RAE. Of particular interest to our study is the Galatea effect (see Merton, 1957) included in the three principal social agents model since the athletes in our study were asked a number of preliminary questions associated with the existence/nonexistence of the RAE.

Wattie et al. (2015), using Newell's (1986) constraints-based model, proposed another RAE theoretical framework. According to Newell, there are three interacting types of constraints responsible for optimal coordination and control of an activity: individual constraints, task constraints, and environmental constraints. Wattie and colleagues associated these three constraints with the RAE and discussed them from a developmental systems theory perspective. They argued that the interrelated associations between the discussed three constraints have the potential to influence the RAE and its appearance/non-appearance in female and male athletes involved in individual and team sports.

However, a number of studies did not find a RAE, namely, that those athletes who were born early in the competition year had a higher representation than those who were born late in that year (e.g., Baker et al., 2009; MacDonald et al., 2009; Barrenetxea-Garcia et al., 2018; Jones et al., 2018). Of particular interest to the objectives of the current study are two studies in which RAE data were collected in elite male and female Israeli ballplayers. In one study (Lidor et al., 2010), the data were collected from 521 male athletes who played basketball (*n* = 68), team handball (*n* = 161), soccer (*n* = 209), and volleyball (*n* = 83) in Division 1 (the highest division of competitive sports in Israel) clubs in Israel. In the second study (Lidor et al., 2014), the RAE data were collected from Israeli female ball players–389 female players playing for various Division 1 ball clubs: 46 basketball players, 107 team handball players, 156 soccer players, and 80 volleyball players. These two studies did not find a significant RAE in these athletes.

Lidor and colleagues proposed two explanations for the lack of RAEs in these elite ballplayers (Lidor et al., 2010, 2014). The first explanation was associated with the small size of the population in the investigated country, Israel, and, consequently, the relatively low number of children interested in participating in sports activities. In the last few years (2017–2019), the Central Bureau of Statistics in Israel has made an effort to present data on the number of children and youth (ages 7–18 years) who participate in different sports activities (Central Bureau of Statistics, 2019). However, despite this effort, no solid information has been made available on the number of children under the age of 14 who engage in competitive sports activities in the country.

Since only a relatively small number of children in Israel select sports as their preferred activity, the selection process of the coaches is probably more flexible compared with coaches in other countries. That is to say, not only the strongest children in the given year are selected to be on the teams but also those who may be less strong in their abilities but possess a potential for future success. It was suggested that, because of Israel's small population and limited opportunities for participation in sports, children are not selected or “de-selected” for participation in sports according to their physical maturity.

The second explanation is the “open door” policy adopted by most of the clubs in the country. Since relatively few children participate in competitive sports activities, ball clubs struggle to recruit children for their specific sport. In essence, each club is competing with the other clubs to recruit more children. In general, the policy of the clubs is to enable a child who shows an interest in a particular ball game to join the team. The assumption of the sport policymakers is that those who are talented and motivated to excel will remain in the program for a longer period of time, and thus their abilities and skills will improve. In contrast, those who have less talent and are not highly motivated to achieve will eventually drop out.

The data discussed in Lidor et al. (2010, 2014) were collected on adult ballplayers who had reached the highest level of competitive sport in one local sports system. In line with the recommendations of Baker et al. (2018), in order to enable the analysis of long-term developmental trends associated with a local sports system, data on various environmental variables associated with this system—such as the RAE—should be collected continuously and compared across different periods of time. In the current study, we analyzed the RAE of young athletes in sports programs similar to the programs from which the RAE data were collected in the elite ballplayers in the two studies of Lidor et al. (2010, 2014). It was our aim to perform a follow-up analysis on these programs and to determine the existence/nonexistence of the RAE in young athletes, a decade after the initial data on this environmental variable were collected.

In addition, up to now, RAE data were obtained from young and adult female and male athletes without “listening” to the athletes' perceptions of this effect as it relates to their own athletic development, if indeed the effect does exist. It would be beneficial for coaches and policymakers to gather RAE-related information from the athletes themselves. It has recently been argued by Baker et al. (2020) that “To date, there has been scant research that has employed qualitative methodologies, leaving a gap in our understanding of how athletes perceive RAEs…” (p. 158). It is assumed that qualitative information that is collected from athletes can also assist coaches, policymakers, and sports directors to better cope with the asymmetrical distribution of athletes who were born early in the year compared with those who were born late in the year.

In only one study (Sherman and Hancock, 2016) was qualitative information gathered from athletes (ten 14- to 15-year-old competitive youth hockey players) and their parents on their awareness and perceptions of the RAE. It was found that none of the athletes had prior knowledge of the RAE, while most of the parents were aware of this phenomenon. In addition, the athletes perceived that players were often selected to teams based on their physical characteristics rather than athletic ability. The parents believed that the RAE was a result of relatively older athletes being more physically mature than the relatively younger athletes on the team. In another qualitative study (Andronikos et al., 2015), seven experts in the field of talent identification and development were interviewed on the existence, mechanisms, and possible solutions to RAEs. Inductive analysis of the data showed that while there was mixed evidence for the RAEs across sports, the eradication of RAEs was attributed to controllable features of the developmental environment. The factors discussed included the structure of categories used to group athletes within the sport (e.g., age and weight), recognition, and prioritization of long-term development over “short-term win focus.”

Therefore, the purpose of our study was threefold: (a) to calculate the RAE in young Israeli elite athletes in both individual and team sports; (b) to study the athletes' initial approach toward the contribution of their date of birth to early success in sport, in light of the three principal social agents model proposed by Hancock et al. (2013), and particularly of the Galatea effect; and (c) to compare the RAE findings of this study with those of two previous studies on elite male (Lidor et al., 2010) and female (Lidor et al., 2014) Israeli ballplayers. In the current study, we adopted a follow-up approach—examining the RAE in athletes who participated in sports programs similar to the ones studied 10 years ago, as well as adding one more dimension to the collection of the RAE data: how the athletes associated their date of birth with various aspects of their training program. While in previous studies on the RAE in Israeli elite athletes only team sports were examined, in the current study, we analyzed the existence of the effect also in individual sports.

We assumed that the findings of the RAE phenomena in young athletes might differ after a 1-decade period due to new initiations or modifications in sport policy made by the local sports authorities. In Israel, for example, a national sports program was established in 2013, Sport's Flowers (see https://www.gov.il/en/departments/ministry_of_culture_and_sport), aimed at increasing the number of young female and male athletes who participate in organized sports activities. Such national initiations have the potential to influence a number of factors associated with early talent development in sports, among them the RAE.

## Method

### Participants

The RAE was assessed in 1,397 athletes aged 14–18 years (mean age = 16.37 years, SD = 1.54); among them are 390 females (mean age = 16.28 years, SD = 1.57) and 1,007 males (mean age = 16.40 years, SD = 1.53). The athletes competed in 10 different sports: 5 individual (gymnastics, judo, swimming, tennis, and track and field) and 5 team (basketball, soccer, team handball, volleyball, and water polo) sports. The numbers of male and female athletes in each sport are presented in Tables 1, 2, respectively.

**Table 1:** Distribution of the male athletes' birth month and the results of the statistical analyses.

**Quartile distribution (CD)**	**Quartile and number and percentage of players**								
	**Q1:** **24.5%**	**Q2:** **23.3%**	**Q3:** **26.4%**	**Q4:** **25.9%**	**Total**	**χ^**2**^ (*df* = 3)**	***p***	**Cramer's *V***	**Q1 *vs*. Q4:** **OR (95% CI)**
Gymnastics (%)	8 (44.44)	4 (22.22)	3 (16.67)	3 (16.67)	18	4.17	0.24	0.34	2.82 (0.44–18.08)
Expected	4.41	4.19	4.75	4.65					
Judo (%)	53 (30.29)	44 (25.14)	46 (26.29)	32 (18.29)	175	6.57	0.09	0.14	2.82 (0.95–3.21)
Expected	42.86	40.70	46.09	45.285					
Swimming (%)	38 (36.54)	26 (25.00)	24 (23.08)	16 (15.38)	104	11.16	0.01^*^	0.23	2.51^*^ (1.13–5.56)
Expected	25.47	24.19	27.45	26.89					
Residual	12.53	1.91	−3.45	−10.89					
Tennis (%)	41 (32.28)	26 (20.47)	30 (23.62)	30 (23.62)	127	4.2	0.24	0.13	1.44 (0.73–2.85)
Expected	31.10	29.54	33.52	32.84					
Track and field (%)	7 (12.07)	18 (31.03)	18 (31.03)	15 (25.86)	58	5.64	0.13	0.22	0.49 (0.16–1.56)
Expected	14.20	13.49	15.30	15.00					
Basketball (%)	54 (34.18)	40 (25.32)	35 (22.15)	29 (18.35)	158	10.86	0.01^*^	0.19	1.97^*^ (1.05–3.69)
Expected	38.70	36.75	41.70	40.86					
Residual	15.30	3.25	−6.70	−11.86					
Soccer (%)	87 (40.47)	41 (19.07)	47 (21.86)	40 (18.60)	215	30.14	0.00^*^	0.26	2.30^*^ (1.35–3.91)
Expected	52.66	50.00	56.75	55.60					
Residual	35.34	−9.00	−9.75	−15.60					
Team handball (%)	16 (31.37)	18 (35.29)	6 (11.76)	11 (21.57)	51	8.66	0.03^*^	0.29	1.54 (0.52–4.57)
Expected	12.49	11.86	13.46	13.19					
Residual	3.51	6.14	−7.46	−2.19					
Water polo (%)	5 (14.29)	12 (34.29)	10 (28.57)	8 (22.86)	35	3.50	0.32	0.22	0.66 (0.15–2.83)
Expected	8.57	8.14	9.24	9.05					
Volleyball (%)	18 (27.27)	16 (24.24)	15 (22.73)	17 (25.76)	66	0.57	0.90	0.07	1.12 (0.43–2.89)
Expected	16.16	15.35	17.42	17.07					
*Total*	327	245	234	201	1,007				

*Q1: January–March; Q2: April–June; Q3: July–September; Q4: October–December*.

*CD, census data*.

* *p ≤ 0.05*.

**Table 2:** Distribution of the female athletes' birth month and the results of the statistical analyses.

	**Quartile and number and % of players**								
**Quartile Distribution (CD)**	**Q1** **(24.5%)**	**Q2** **(23.3%)**	**Q3** **(26.4%)**	**Q4** **(25.9%)**	**Total**	*****χ^2^***(*df* = 3)**	***P***	**Cramer's V**	**Q1 vs. Q4** **OR (95% CI)**
Gymnastics	11 (23.91%) 11.27	11 (23.91%) 10.70	11 (23.91%) 12.14	13 (28.26%) 11.89	46	0.22	0.97	0.05	089 (0.29; 2.81)
Judo	11 (21.57%) 12.49	16 (31.37%) 11.86	11 (21.57%) 13.46	13 (25.49%) 13.19	51	2.07	0.56	0.14	0.89 (0.29; 2.71)
Swimming	16 (32.00%) 12.25	15 (30.00%) 11.63	11 (22.00%) 13.20	8 (16.00%) 12.93	50	4.37	0.22	0.21	2.11 (0.66; 6.69)
Tennis	14 (26.92%) 12.74	13 (25.00%) 12.09	13 (25.00%) 13.72	12 (23.08%) 13.45	52	0.39	0.94	0.60	1.23 (0.42; 3.65)
Track and Field	6 (22.22%) 6.61	9 (33.33%) 6.28	9 (33.33%) 7.13	3 (11.11%) 6.98	27	3.99	0.26	0.38	2.11 (0.37; 12.12)
Basketball	22 (28.95%) 18.61	18 (23.68%) 17.68	22 (28.95%) 20.06	14 (18.42%) 19.65	76	2.44	0.48	0.13	1.66 (0.66; 4.17)
Soccer	8 (38.10%) 5.14	4 (19.05%) 4.88	7 (33.33%) 5.54	2 (9.52%) 5.43	21	4.30	0.23	0.32	4.22 (0.59; 30.09)
Team Handball	13 (37.14%) 8.57	9 (25.71%) 8.14	7 (20.00%) 9.24	6 (17.14%) 9.05	35	3.95	27.0	24.0	2.29 (60; 8.79)
Water Polo	0 (0.00%) -	0 (0.00%) -	1 (33.33%) -	2 (66.67%) -	3	-	-	-	-
Volleyball	10 (34.48%) 7.10	6 (20.69%) 6.74	11 (37.93%) 7.65	2 (6.90%) 7.50	29	6.76	0.80	0.34	5.28 (0.85; 32.99)
Total	111	101	103	75	390				

*Q1: January–March; Q2: April–June; Q3: July–September; Q4: October–December*.

*CD, census data*.

We are aware of the fact that the number of female athletes who participated in the current study is low (*n* = 390) compared to the number of male athletes. In our previous study on adult female ball players, the number of the female participants was similar (*n* = 389); however, this represented individuals from only four sports activities. The low number of female participants in the current study reflects the limited number of young active female athletes in Israel. For example, only 19% of the active athletes 13–18 years of age are females (see Central Bureau of Statistics, 2019). This small sample creates a dilemma: on the one hand, due to this small sample size of female athletes, the probability of revealing a significant finding in the RAE data is low. On the other hand, in order to discuss theoretical and practical implications of the RAE from the local sport system's perspective, the existence (or nonexistence) of the RAE should be analyzed. It was our preference to analyze the existence of the RAE in female athletes, taking into account the restrictions of the small sample size.

We selected 14 (as the early age) to 18 (as the late age) year-old athletes because these age categories represent two critical transitional phases in the early career of a young competitive athlete. The age of 14 years is considered to be a turning point from the developmental years to the specialization years (Côté et al., 2006), and the age of 18 years is considered to be a transitional stage from being part of a youth sports program to becoming a member of a professional/semi-professional adult sports program (Lidor et al., 2010). The athletes in this study participated in between four and six sessions of practice on a weekly basis, depending upon the given sport. In addition, they regularly competed in organized leagues and tournaments.

Typically, sports programs in Israel allow registration at the age of 8 years. The participants in our study had at least 6 years' experience in training and competition. However, in a number of sports, such as gymnastics, swimming, and soccer, children can enter the sports program even earlier than the age of 8 years, and thus some of them had gone through at least 9 years of training and competition. Therefore, the participants in our study were aged 14–18 years and could have participated in 6–9 years of activity in their selected sports. The study was approved by the ethics committee of the Academic College at Wingate.

### RAE Calculations and Perceptions Related to the RAE

Since the mean age of the participants (females and males) in our study was 16.37 years (SD = 1.54) and the majority of the data in our study were collected in 2016, census data from the year 2000 were used. In this year, the distribution of the birth dates per quartile was as follows: Q1 (January–March), 24.5%; Q2 (April–June), 23.3%; Q3 (July–September), 26.4%; and Q4 (October–December), 25.9%. This distribution of the birth dates per quartile in the year 2000 was used as the expected distribution for the chi-square tests. Based on these data, we can observe that a similar distribution of birth dates across quartiles existed in this year. The comparison of the RAE data collected in the current study was based on this observation.

The athletes who participated in the current study were asked to answer in writing two closed questions related to the RAE. In question 1, the athletes were asked whether they felt that their birth date (the month they were born in the year) had an influence (positive/negative) on the way they were able to develop their athletic abilities/skills. The athletes were given two options: “yes” or “no.” They had to select one of the two options and to encircle the selected one. They were not asked to add any more written information.

In question 2, the athletes were asked whether they felt that they had any strengths/limitations in one or more of the following four pillars of the training program—physical, cognitive, emotional, and social—compared to their peers in the sports program. We selected these four pillars because they are considered the foundation for any sports program aimed at improving abilities/skills in a young athlete (see, e.g., Bompa and Buzzichelli, 2018; Bompa et al., 2019).

The athletes were provided with written information about the meaning of the four selected pillars and their association with sport-related abilities and skills. The athletes were informed that (a) the term *physical pillar* is associated with technique, as well as athletic abilities such as agility, coordination, flexibility, and speed; (b) the term *cognitive pillar* is associated with processes, such as decision making and game understanding (for the players who played team sports); (c) the term *emotional pillar* is related to internal and external motivation, self-confidence, and self-discipline; and (d) the term *social pillar* is related to leadership and team cohesion.

In question 2, the athletes were given four options: “physical pillar,” “cognitive pillar,” “emotional pillar,” and “social pillar.” They were asked two questions about these pillars: (a) Do you feel that you have any strengths in one or more of the following four pillars of the training program—physical, cognitive, emotional, and social—compared to your peers in the sports program?, and (b) Do you feel that you have any limitations in one or more of the following four pillars of the training program—physical, cognitive, emotional, and social—compared to your peers in the sports program? The athletes had to select one or more of the four options and to encircle the selected one/s for each question. They were not asked to add any more written information.

### Procedure

Information about an athlete's birth date, gender, and his or her type of sport were collected *via* questionnaires, which also included the two RAE-related questions. The questionnaires were administered to the athletes by their coaches. Each director of the sports program where the athletes who participated in the study practiced received a letter providing the background and objectives of the study. All of the directors approved the study. In addition, informed consent was obtained from the parents of the participants. After approval was obtained, the second author approached the coaches of the athletes and sent them the questionnaires *via* electronic mail.

All of the coaches who were approached (*n* = 68; 14 females and 54 males, all certified by their sports federations) agreed to gather the required information from their athletes. The coaches administered the questionnaires, collected them, and sent them back to the researchers. The administration of the questionnaires to the athletes was performed manually, so that any questions relating to question 1 and question 2 that were raised by the athletes could be addressed directly by the coaches. Prior to the administration of the questionnaires, the coaches were provided with detailed information about the study. They were also prepared to answer other questions that could have been raised by the athletes, particularly those related to their birth date (early or late in their cohort year) and the four pillars (e.g., the meaning of the physical pillar, the cognitive pillar, etc.).

We collected the data on the RAE *via* such a process since information on the birth dates of the athletes could not be obtained from the official websites of the relevant sports federations or the specific sports programs. We had also faced this challenge in previous studies examining the RAE in elite male and female ballplayers in Israel (Lidor et al., 2010, 2014).

### Data Analysis

A chi-square (χ^2^) test was performed to determine the significance of deviations for the expected number of birth dates in each quartile of the selected year. The chi-square test does not reveal the magnitude of difference between the quartiles' distributions for the significant chi-square outputs. Therefore, in line with the RAE analyses of Kelly et al. (2020), Cramer's *V* was also performed. Odds ratios (ORs) and 95% confidence intervals (CIs) were also used to compare the quartiles for the observed and expected distributions.

The birth date of each athlete in each sport was recorded to represent his or her birth quartile (Q). Four quartiles were designated: Q1 = January–March; Q2 = April–June; Q3 = July–September; and Q4 = October–December. Athletes who were born in Q1 were considered to be relatively older than those who were born in Q4. Chi-square tests were also used to analyze the data collected from question 1 and question 2. The analysis was performed separately for the male and female athletes and for the individual and team sports.

## Results

Results are presented separately for the RAE, question 1, and question 2. As indicated previously, the sample size of the female athletes was relatively small. However, it was our aim to analyze their data separately in order to obtain information on each gender and on each sport. In this way, we are able to strengthen our understanding of the existence of the RAE in the various sports programs available to female and male children and youth in the country.

### Relative Age Effect

#### Male Athletes

The frequency and percentage distribution of the male athletes' birth months and the results of the χ^2^ test are presented in Table 1. The χ^2^ test showed that the RAE was significant in male athletes in one individual sport, swimming (*p* < 0.01), and in three team sports, basketball (*p* < 0.01), soccer (*p* < 0.001), and team handball (*p* < 0.03). In swimming, basketball, and soccer, those who were born in Q1 had a higher representation in the elite youth programs compared to those who were born in Q2, Q3, and Q4. In team handball, those who were born in Q2 had a higher representation in the elite youth program compared to those who were born in Q1, Q3, and Q4. It can be observed in the abovementioned four sports that the number of athletes who were born in the early phases of the year was significantly higher than the number of those who were born in the late phases of the year.

#### Female Athletes

The frequency and percentage distribution of the female athletes' birth months and the results of the χ^2^ test are presented in Table 2. The RAE was not found to be significant among the female athletes in any of the analyzed sports. That is to say, a similar number of individuals from each quartile were selected to participate in the designated sport programs.

### Question 1

All the athletes (females and males) who participated in the study answered question 1. Among the male athletes, the majority (79.2%) reported that they did not feel that their date of birth influenced their athletic development, while only 20.8% perceived it as a contributing factor [χ^2^ (1) = 343.18, *p* < 0.001]. More specifically, 77.2% of the male athletes in the individual sports and 81% in the team sports reported that they did not perceive their birth date as an influential factor in their athletic development.

Similar findings were found for the female athletes. The majority of the female athletes (88.2%) did not perceive their birth date as an influential factor in their sports development, leaving only 11.8% who perceived their birth date as being influential [χ^2^ (1) = 226.76, *p* < 0.001]. More specifically, 85.3% of the female athletes in the individual sports and 92.1% in the team sports reported that they did not perceive their birth date as a contributing factor to their athletic development.

A comparison between the male and female athletes revealed that a greater portion of the males (20.8%) perceived their date of birth as a contributing factor to their success than did the females (11.8%) [χ^2^ (1) = 15.6, *p* < 0.001].

### Question 2

Among the male athletes, 840 out of 1,097 (77%) answered question 2. Among the female athletes, 310 out of 390 (79.4%) who participated in the study answered this question. The data (number of athletes, percentage of athletes, χ^2^ values, and level of significance) on how the male and female athletes who perceived themselves as having strengths in the four pillars of the training program are presented per quartile in Tables 3, 4, respectively.

**Table 3:** Distribution across quartiles of the male athletes who felt they had strengths in the four pillars.

**Sport**	**Pillar**	**Q1**		**Q2**		**Q3**		**Q4**		**Total**		**χ^**2**^**	***p***
		***N***	**%**	***N***	**%**	***N***	**%**	***N***	**%**	***N***	**%**		
Individual	Physical	64	35.4	46	25.4	37	20.4	34	18.8	181	37.4	12.08	0.01^*^
	Cognitive	15	38.5	8	20.5	10	25.6	6	15.4	39	8.1	4.59	0.20
	Emotional	16	33.3	10	20.8	13	27.1	9	18.8	48	9.9	2.50	0.48
	Social	0	0.0	1	25.0	2	50.0	1	25.0	4	0.8	2.00	0.57
Team	Physical	73	36.3	53	26.4	42	20.9	33	16.4	201	38.1	17.73	0.00^*^
	Cognitive	28	35.9	9	11.5	16	20.5	25	32.1	78	14.8	11.54	0.01^*^
	Emotional	14	31.8	8	18.2	12	27.3	10	22.7	44	8.3	1.82	0.61
	Social	10	45.5	4	18.2	4	18.2	4	18.2	22	4.2	4.91	0.18

* *p < 0.05*.

**Table 4:** Distribution across quartiles of the female athletes who felt they had strengths in the four pillars.

**Sport**	**Pillar**	**Q1**		**Q2**		**Q3**		**Q4**		**Total**		**χ^**2**^**	***p***
		***N***	**%**	***N***	**%**	***N***	**%**	***N***	**%**	***N***	**%**		
Individual	Physical	21	31.8	19	28.8	15	22.7	11	16.7	66	29.2	3.58	0.31
	Cognitive	2	11.8	7	41.2	6	35.3	2	11.8	17	7.5	4.88	0.18
	Emotional	6	31.6	7	36.8	5	26.3	1	5.3	19	8.4	4.37	0.22
	Social	1	20.0	3	60.0	1	20.0	0	0.0	5	2.2	3.80	0.28
Team	Physical	23	32.9	15	21.4	21	30.0	11	15.7	70	42.7	5.20	0.16
	Cognitive	3	27.3	3	27.3	3	27.3	2	18.2	11	6.7	0.27	0.97
	Emotional	6	40.0	2	13.3	4	26.7	3	20.0	15	9.1	2.33	0.51
	Social	2	50.0	1	25.0	0	0.0	1	25.0	4	2.4	2.00	0.57

Five main findings emerged from the data analyses. Firstly, the athletes preferred sharing their perspectives about their strengths in the four pillars rather than about their limitations. More specifically, among the 840 male athletes who answered this question, 617 (73%) shared their feelings about their strengths and only 223 (27%) added information about their limitations. Similar findings were obtained for the female athletes: among the 310 athletes who answered this question, 207 (67%) reported about their strengths and 103 (33%) about their limitations.

Secondly, among the four pillars of the training program, more male and female athletes in both individual [181 (37.4%) and 66 (29.2%), respectively] and team [201 (38.1%) and 70 (42.7%), respectively] sports associated their strengths with the physical pillar rather than with any of the other pillars. Similar findings were obtained for those athletes who provided information about having limitations: 161 out of 223 (72%) male athletes and 77 out of 103 (74%) female athletes related their limitations to the physical pillar.

Thirdly, for the male athletes in individual sports, it was found that those who were born in Q1 (*n* = 64; 35.4%) and Q2 (*n* = 46; 25.4%) believed that they were superior to their peers who were born in Q3 (*n* = 37; 20.4%) and Q4 (*n* = 34; 18.8%). More specifically, those who were born early in the year felt that they were superior in their techniques in comparison to their counterparts who were born late in the year. In addition, those who were born in Q1 and Q2 perceived themselves as better athletes with regard to their agility, coordination, flexibility, and speed than their peers who were born in Q3 and Q4.

Fourthly, a similar observation as the one made for the male athletes in individual sports can be made for the male athletes in team sports. More athletes who were born in Q1 (*n* = 73; 36.3%) and Q2 (*n* = 53; 26.4%) believed that they had greater physical strengths compared with those who were born in Q3 (*n* = 42; 20.9%) and Q4 (*n* = 33; 16.4%). In addition, it was found for the male athletes in team sports that more athletes who were born in Q1 (*n* = 28; 35.9%) and Q4 (*n* = 25; 32.1%) believed that they had greater cognitive strengths compared to those who were born in Q2 (*n* = 9; 11.5%) and Q3 (*n* = 16; 20.54%). In other words, those who were born early in the year (Q1), as well as those who were born in the last phase of the year (Q4), felt that they were better in their decision-making and game-understanding processes than those who were born in Q2 and Q3.

Lastly, no significant differences were found in how the female athletes who were born in any of the four quartiles perceived their strengths/limitations in the four pillars of the training program.

## Discussion

Three main findings emerged from the current study. Firstly, the RAE was found to be significant only in the male athletes and in only four out of the 10 investigated sports. More specifically, the effect was found in swimming (an individual sport) and in basketball, soccer, and team handball (team sports). In the female athletes, the RAE did not exist. Secondly, both female and male athletes did not feel that their birth date had any relation to their athletic achievement. Thirdly, when asked whether they felt that they had any strengths/limitations in the four basic pillars of the training program—physical, cognitive, emotional, and social—compared to their peers in the program, most of the athletes (both females and males) related to their strengths rather than to their limitations. Specifically, male athletes who were born in Q1 and Q2 felt that they had more physical strengths compared to those who were born in Q3 and Q4. Those who were born in Q1 among the male athletes believed that they also had more cognitive strengths compared to their peers who were born in Q2 and Q3. However, those who were born in Q4 felt the same, namely that they had more cognitive strengths compared to those who were born in Q2 and Q3.

### Relative Age Effect

The RAE data obtained in the current study are partially in line with those reported in previous studies. On the one hand, the finding that RAE was present in males in four sports is in line with previous findings (e.g., Côté et al., 2006; Baker and Logan, 2007; Schorer et al., 2009). On the other hand, the finding that the RAE did not exist in the male athletes in six out of the 10 analyzed sports and the finding that the RAE was not found in the female athletes altogether are not in line with the majority of the previous findings on the RAE (see reviews by Cobley et al., 2009; MacDonald and Baker, 2013; Sierra-Díaz et al., 2017; Smith et al., 2018). However, the nonexistence of the RAE in most of the sports examined in the current study (a combined 16 out of 20 activities for both female and male athletes) is in line with the findings of RAEs reported in earlier studies on Israeli elite sport performers (Lidor et al., 2010, 2014).

We offer two explanations for the RAE data obtained in our study. Firstly, in most of the analyzed sports where the RAE did not exist, the coaches who recruited children to the sports programs continued to implement the “open door” policy, as described by Lidor et al. (2010, 2014). According to this policy, children who are selected to join a sports program in Israel are encouraged to continue their sports experience, even though some of them do not demonstrate the physical attributes required to achieve a high level of proficiency in sports. It is assumed that this policy enables those who are considered to be late bloomers to continue their sports participation and become part of an effective training environment with highly qualified coaches.

The second explanation is associated with the popularity of ballgame activities in Israel. More specifically, soccer and basketball—followed by volleyball and team handball—are considered to be the most popular sports in Israel (see Lidor and Blumenstein, 2012). For example, the Central Bureau of Statistics (2019) reported that, in 2017–2018, 48,799 children and youth (ages = 12–17 years) were active in ballgame activities in Israel, about 4% of the total population at this age. Among them, 40.9% (*n* = 29,808) were soccer players and 26,697 (36.8%) were basketball players. The rest of the children and youth (22%) were active in 16 different ballgame activities (including team handball and volleyball). Therefore, due to the fact that there are far more children in basketball and soccer who want to be part of a talent development program than in other sports, basketball and soccer coaches do not adopt the “open door” policy as is done in the other sports, but instead implement a more cautious approach in their early selection processes. That is to say, coaches select those who are more fit physically for contact sports such as soccer and basketball. This is also true for the game of team handball, where the RAE was found to be significant in the male athletes, although the number of children who are interested in playing team handball is much smaller than those who have an interest in soccer and basketball.

In the less popular sports, coaches are somewhat obliged to select children who are not necessarily advanced in their physical development since there are only a small number of children who show interest in these sports contexts. It has already been suggested that the lack of competition can serve as a moderator for the RAE (Musch and Grondin, 2001; Schorer et al., 2009).

The sport of swimming seems to be an interesting RAE case in our study. Swimming is considered a minor individual sport in Israel, but the RAE did exist in the male swimmers who participated in our study. In the years 2017–2018, there were only 321 active competitive swimmers in Israel between the ages of 7 and 24 years. About 55% of them were active between the ages of 7 and 11 years and about 43% between the ages of 12 and 17 years. Although not many children selected swimming as their preferred sport, we assume that the RAE did exist in the young male swimmers due to the fact that the time allocated to practice sessions in the community swimming pools is limited for competitive swimmers, and therefore the coaches are obliged to select only the best in their age group to be included in the team.

### Perceptions About Issues Related to the RAE

Up to now, only one study has examined the perceptions of athletes concerning various aspects associated with the RAE (Sherman and Hancock, 2016). Since only limited information was collected in questions 1 and 2, we adopted a cautious approach in our attempts to interpret the athletes' responses. Two observations can be made based on the analysis of the athletes' responses. Firstly, most of the athletes did not think that their birth date is associated with athletic development; those who were born early in the year did not refer to it as a contributing factor to their success, while those who were born late in the year did not relate to it as an obstacle to achieving success. In question 1, the athletes were not asked about their prior knowledge of the RAE. It is possible that they were familiar with the term, or perhaps they were not. We assumed that they were not familiar with the specific term RAE; however, after 6–9 years of experience in their selected sports, the athletes probably could relate to their date of birth (early or late in the year) as a potential variable associated with their athletic achievements. It is suggested that, in future studies on the RAE, the effect should be related to the athletes' early sport development.

This finding is somewhat in line with the finding that emerged from Sherman and Hancock's (2016) study, namely that the young competitive hockey players had no prior knowledge of the RAE. In both studies, the young athletes (14–18 years old in our study and 14–15 years old in Sherman and Hancock's study) did not consider the RAE as a contributing/interfering variable to their athletic performance. Taking into account (a) the finding that the RAE was found to be significant in only four sports in the male athletes and (b) the finding that the athletes themselves did not value this effect as a contributing factor to their success, we can speculate that the coaches did implement an “open door” approach in the early phases of talent selection. It appears that the coaches provided children with a real opportunity to be part of the sports programs, regardless of whether they were born early or late in the given year.

Secondly, when asked about their strengths/limitations compared to their peers in the sports program, the male athletes who were born early in the year (Q1 and Q2) felt that they had more strengths in the physical pillar of the program compared to their counterparts who were born late in the year (Q3 and Q4). This observation is somewhat contradictory to the feelings the athletes reported in question 1. However, we assume that by introducing the four pillars to the athletes, and not merely providing a general concept of the RAE as we did in question 1, those who were born in Q1 and Q2 could refer more specifically to their strengths/limitations compared to their peers who were born late in the sports program. In addition, we assume that most of the athletes referred mainly to their physical strengths since the physical pillar is the one among the four pillars that is most associated with the athletic/sports domain (Bompa and Buzzichelli, 2018). Support for this finding was found in Sherman and Hancock's (2016) study, where the young players believed that players were often selected to teams based on their physical characteristics rather than on their athletic ability.

We assume that the finding that male athletes who were born early in the year (Q1 and Q2) felt that they exhibited more strengths in the physical pillar of the program compared to their counterparts who were born late in the year (Q3 and Q4) can also explain why a small portion of the male athletes (20.8%) perceived their date of birth as a contributing factor to their success. These athletes, as well as the 12% of the female athletes who also believed that their date of birth was a contributing factor to their success, apparently attributed achieving early success in sports to their more mature physical characteristics (see Sherman and Hancock, 2016). They viewed the physical pillar as the one which contributed the most to early achievements in sports rather than the cognitive, emotional, or the social pillar. This assumption should be further examined in additional qualitative studies.

The male athletes who were born in the different quartiles of a given year also differed in how they perceived their strengths/limitations in the cognitive pillar. However, in this case, not only those who were born early in the year (Q1) but also those who were born late in the year (Q4) felt that they had strengths in the cognitive pillar compared to those who were born in Q2 and Q3. For those who were born in Q1, it can be suggested that they were probably more experienced and therefore also felt superior in the cognitive pillar compared to their peers who were born late in the year. For those who were born in Q4, it can be speculated that they felt superior in the cognitive pillar, perhaps in order to convince themselves that, although they were not as physically strong as the ones who were born early in the year, they still could have positive achievements because they were stronger cognitively than their older peers in the program. Presumably, the latter explanation should be further examined in additional RAE studies.

The finding that male athletes who were born early in the year (Q1 and Q2) felt that they had (a) more strengths in the physical pillar of the program compared to their counterparts who were born late in the year (Q3 and Q4) and (b) more strengths in the cognitive pillar compared to those who were born in Q3 can be explained by the RAE theoretical framework proposed by Hancock et al. (2013). According to this model, the influence athletes have on the RAE is due to the Galatea effect (see Merton, 1957) or to the self-expectations that athletes possess: once expectations are placed upon an individual, that individual typically acts congruently with those expectations. Since social agents, such as coaches and parents, may expect the older athletes to achieve better than those who were born late in the program, the older athletes match themselves up with those expectations. They actually not only believe that they have better abilities than their younger counterparts but also act according to these expectations, thereby achieving a higher level of proficiency in their sport. As argued by Hancock and colleagues “… as athletes buy into those expectations, they raise their self-expectations, affording continued success” (p. 634).

We can use the RAE theoretical model of Hancock et al. (2013) to also explain the finding that those who were born late in the year (Q4) felt, as did those who were born in Q1, that they had strengths in the cognitive pillar compared to those who were born in Q2 and Q3. We assumed that the relatively younger athletes who had succeeded to be part of the competitive sports program also possess high self-expectations since they were already part of the program. Probably, the relevant social agents (e.g., coaches and parents) also expect them to attain success, at least in the cognitive pillar of the program (e.g., making an accurate decision in a ballgame activity).

Since one of our purposes in the current study was to explore how 14- to 18-year-old athletes perceived a number of aspects associated with the RAE, we provided them with a limited number (two) of closed questions. In order to strengthen our understanding of what athletes think about relevant environmental variables associated with their early athletic development, as well as of what other social agents involved in the long-term process of athletic development—such as coaches and parents—think about these variables, additional qualitative information should be collected. For example, performing follow-up interviews with selected athletes from each gender and from each sport has the potential to add value to the findings that emerged from our study. The qualitative approach used by Sherman and Hancock (2016) is promising, and the responses from in-depth interviews with the relevant stakeholders should be gathered and assessed.

### Organizational and Structural Considerations—Looking Back and Forward

The combined RAE findings of the current study (excluding those on four sports in the male athletes), as well as of previous studies on male and female elite ballplayers in Israel (Lidor et al., 2010, 2014), provide support for the nonexistence of the RAE in young and adult Israeli athletes. Due to the unique characteristics of the local sports system, among them that not many children are involved in sports and those who are involved in sports prefer to be part of “big sports” programs such as soccer and basketball, coaches and policymakers cannot be meticulous in their early selection of children to the designated sport programs. In essence, they enable all interested children to join sports programs and therefore provide them with various educational–instructional learning opportunities to acquire sports skills. It can be concluded that the local sports system in Israel has been able to maintain the use of the “open door” approach over the past 10 years.

However, the existence of the RAE in a number of sports in the current study may demonstrate a change in the selection policy in the “big sports” (i.e., basketball, soccer, and team handball) compared to the policy used a decade ago. If the “open door” policy does not currently exist in the “big sports” programs in Israel, at least in the male athletes, policymakers are advised to be aware of this trend and to make efforts to encourage children who are deselected from the “big sports” programs to transfer to other programs where they can develop their abilities/skills as well. If those children who are not initially selected to the “big sports” programs withdraw from all sport participation, then the total number of children who are involved in sports in Israel may become even smaller.

Based on the written responses of the athletes to questions 1 and 2, it might be proposed—although cautiously—that prior to the implementation of imposed actions aimed at preventing/eliminating the RAE, information from the athletes themselves should be gathered in order to study how they perceive this effect. As was found in our study, athletes may not consider their birth date in a given year (early or late) as a contributing/interfering factor to their athletic performance. Indeed, male athletes who were born early in the year felt that they had a larger number of physical and cognitive strengths compared to those who were born early in the year. However, those who were born late in the year did not feel that they had any physical or cognitive limitations compared to those who were born early in the year. In fact, the opposite is true—those who were born in Q4 felt that had more cognitive strengths compared to those who were born in Q2 and Q3 in the same year. We argue that listening to the athletes themselves may aid in searching for effective organizational strategies on how to deal with the RAE, if indeed the effect exists in young athletes.

The main difference between the data that emerged from the previous studies on the RAE in elite athletes in Israel (Lidor et al., 2010, 2014) and the data of the current study is that while in the previous studies the effect was not found, in the current study, the RAE was found to be significant in three team sports and one individual sport in the male athletes. Indeed, this difference may be considered small. However, the existence of the effect in the three most popular sports in the country can provide policymakers with evidence-based data on how to develop current and future sport programs for children and youth. For example, a national sports program was established in 2013 in Israel (see https://www.gov.il/en/departments/ministry_of_culture_and_sport) in order to increase the number of children in sports activities. This national program was composed of various sports, including the most popular ballgame activities in the country: soccer and basketball. It might be more beneficial for policymakers to promote sports programs where the RAE does not exist, thereby enabling more children to join the programs and enjoy early positive sports experiences. It has already been argued that a number of factors, among them the popularity of a given sport, the number of active participants, the importance of physical development, and the competitive level, may influence the magnitude of the RAE (Musch and Grondin, 2001; Romann et al., 2020). Apparently, the RAE that was found in basketball, soccer, and team handball in the current study is associated with the high rates of participation and high selection pressure in these sports.

## Conclusion

In order to validate the existence/nonexistence of the RAE as an environmental factor associated with achieving superiority in sports in a local sports system, data related to this effect should be collected continuously over a long-term period. These data can assist professionals who are involved in processes of early talent selection/development, among them coaches and policymakers, to reflect upon the decisions they are required to make throughout these processes. In addition, the voice of the athletes should also be heard in order to strengthen the interpretation of the numerical RAE data collected from them.

## Data Availability Statement

The raw data supporting the conclusions of this article will be made available by the authors, without undue reservation.

## Ethics Statement

The studies involving human participants were reviewed and approved by The ethics committee of the Academic College at Wingate. Written informed consent to participate in this study was provided by the participants' legal guardian/next of kin.

## Author Contributions

RL and ZM: conceptualization and methodology. ZM: data collection. RL: writing original draft. MA: data analysis. All authors contributed to the article and approved the submitted version.

## Conflict of Interest

The authors declare that the research was conducted in the absence of any commercial or financial relationships that could be construed as a potential conflict of interest.
